# Supplementary material for: BrWAX3, Encoding a β-ketoacyl-CoA Synthase, Plays an Essential Role in Cuticular Wax Biosynthesis in Chinese Cabbage
Source: Int J Mol Sci. 2022 Sep 19;23(18):10938. doi: 10.3390/ijms231810938 (PMC9501823; doi:10.3390/ijms231810938)
Supplement: Supplementary file 1 [file ijms-23-10938-s001.zip › Supplemental materials/Supplemental figures.pptx]

## Slide 1
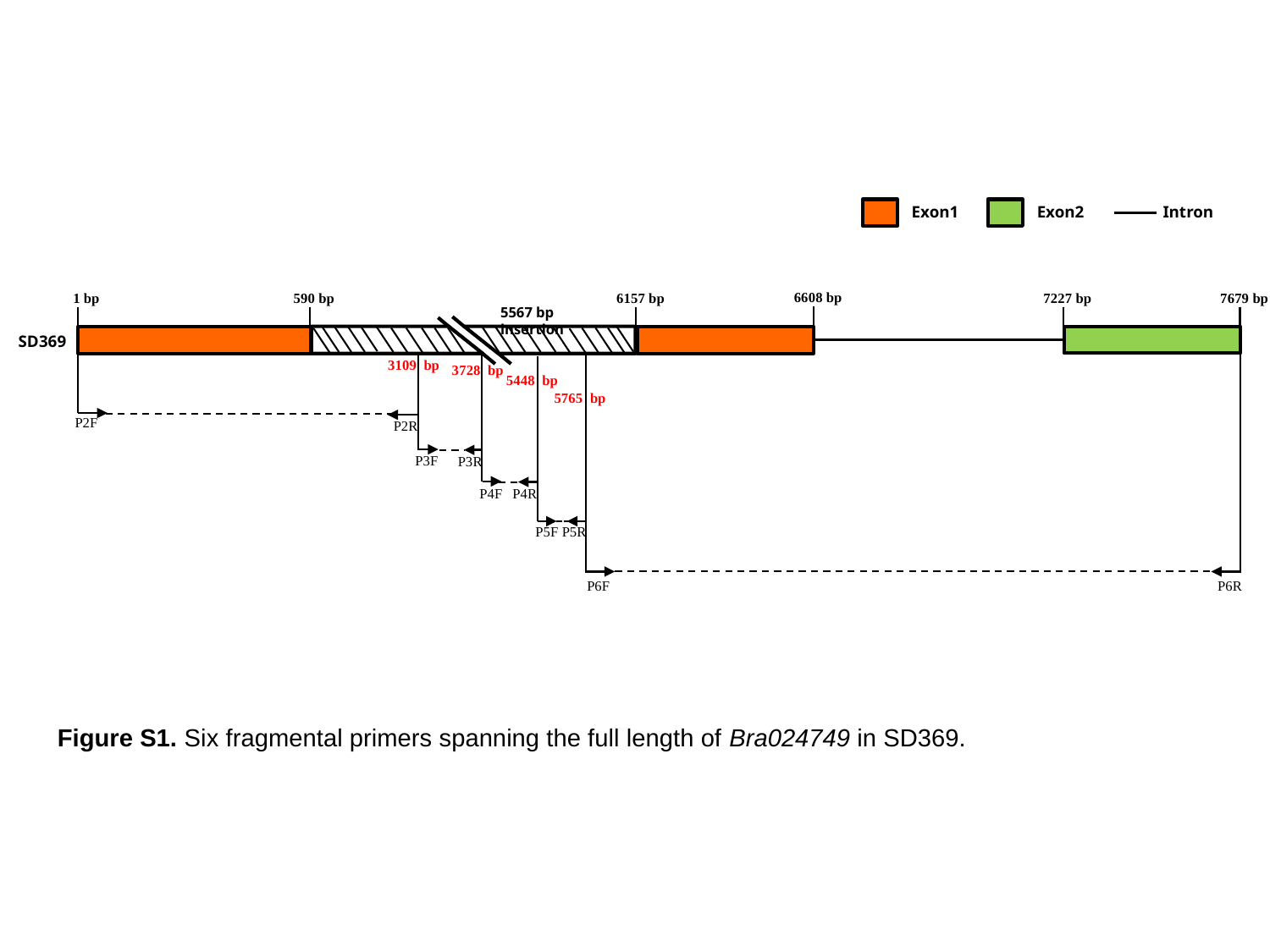

Exon1
Exon2
Intron
6608 bp
1 bp
590 bp
6157 bp
7227 bp
7679 bp
5567 bp insertion
SD369
3109 bp
3728 bp
5448 bp
5765 bp
P2F
P2R
P3F
P3R
P4F
P4R
P5F
P5R
P6F
P6R
Figure S1. Six fragmental primers spanning the full length of Bra024749 in SD369.

## Slide 2
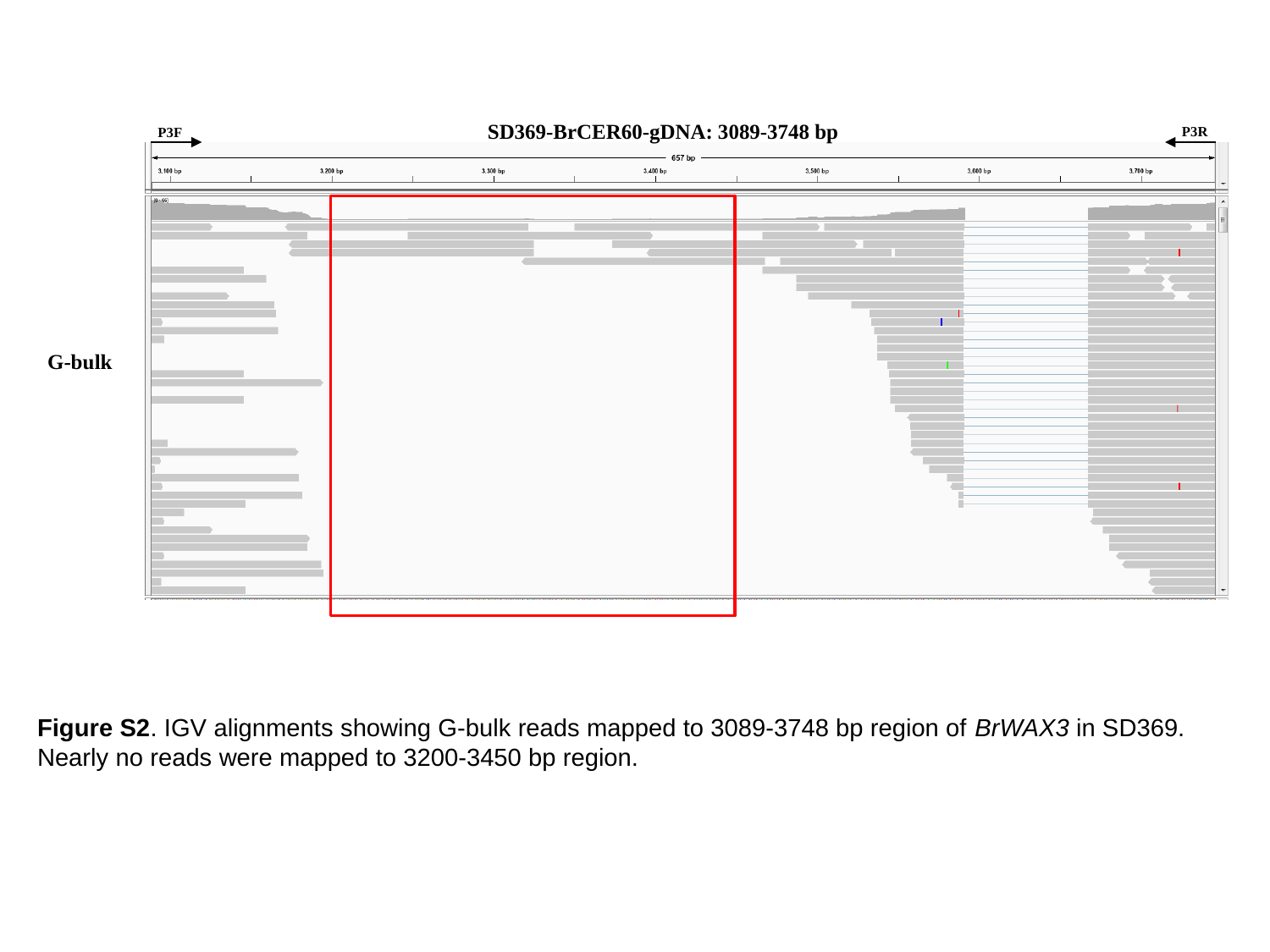

SD369-BrCER60-gDNA: 3089-3748 bp
P3R
P3F
G-bulk
Figure S2. IGV alignments showing G-bulk reads mapped to 3089-3748 bp region of BrWAX3 in SD369. Nearly no reads were mapped to 3200-3450 bp region.

## Slide 3
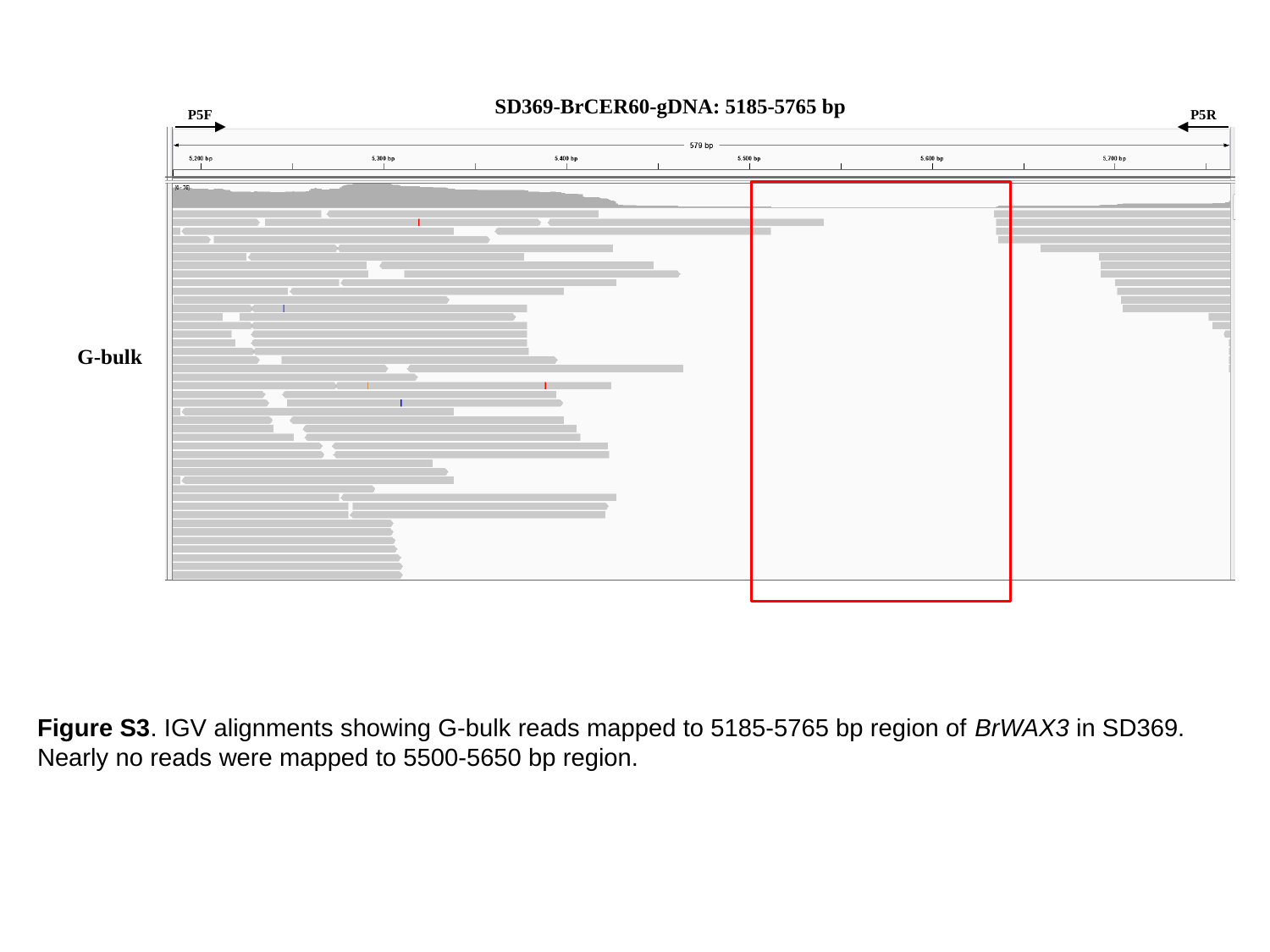

SD369-BrCER60-gDNA: 5185-5765 bp
P5F
P5R
G-bulk
Figure S3. IGV alignments showing G-bulk reads mapped to 5185-5765 bp region of BrWAX3 in SD369. Nearly no reads were mapped to 5500-5650 bp region.

## Slide 4
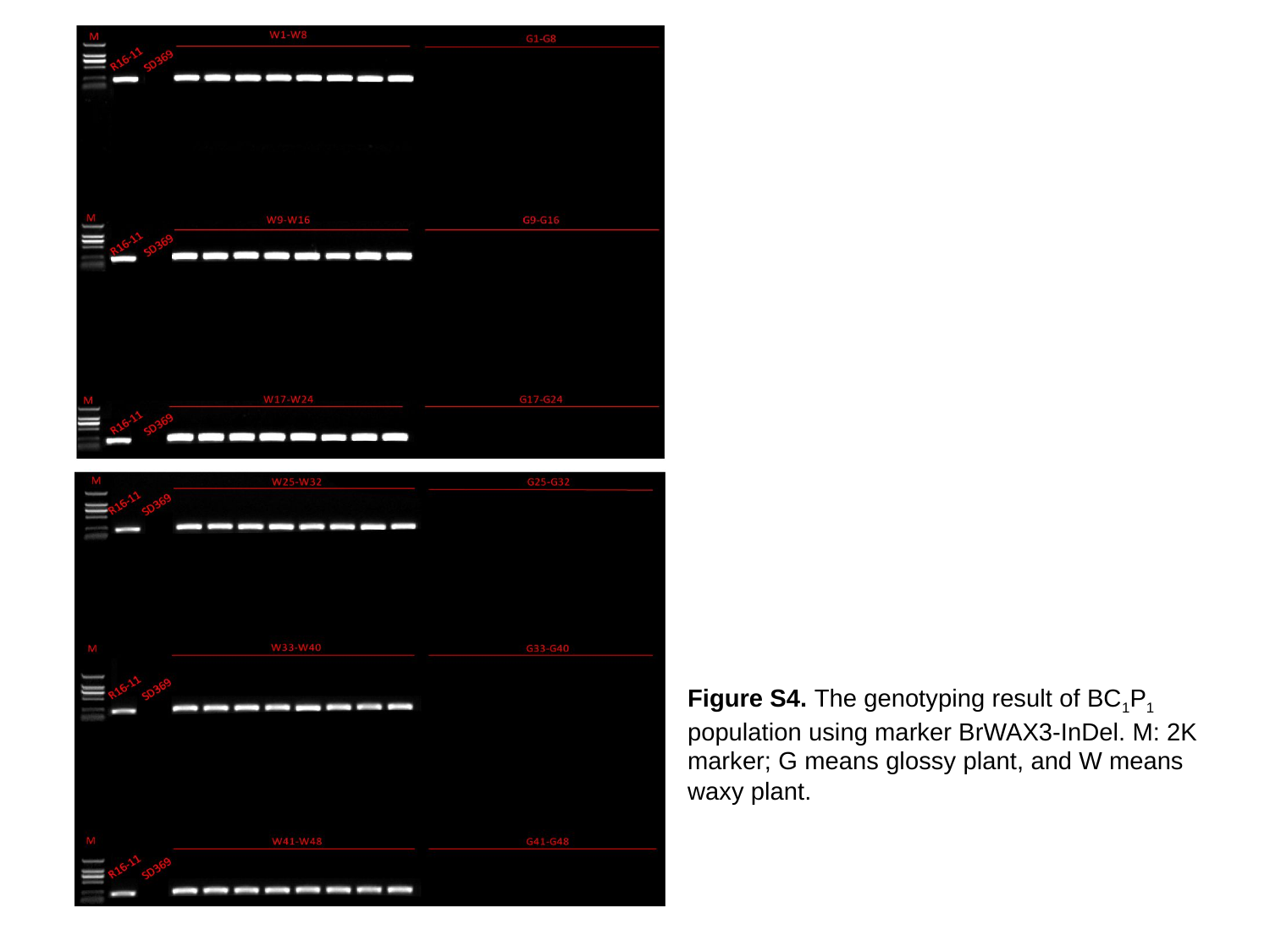

Figure S4. The genotyping result of BC1P1 population using marker BrWAX3-InDel. M: 2K marker; G means glossy plant, and W means waxy plant.

## Slide 5
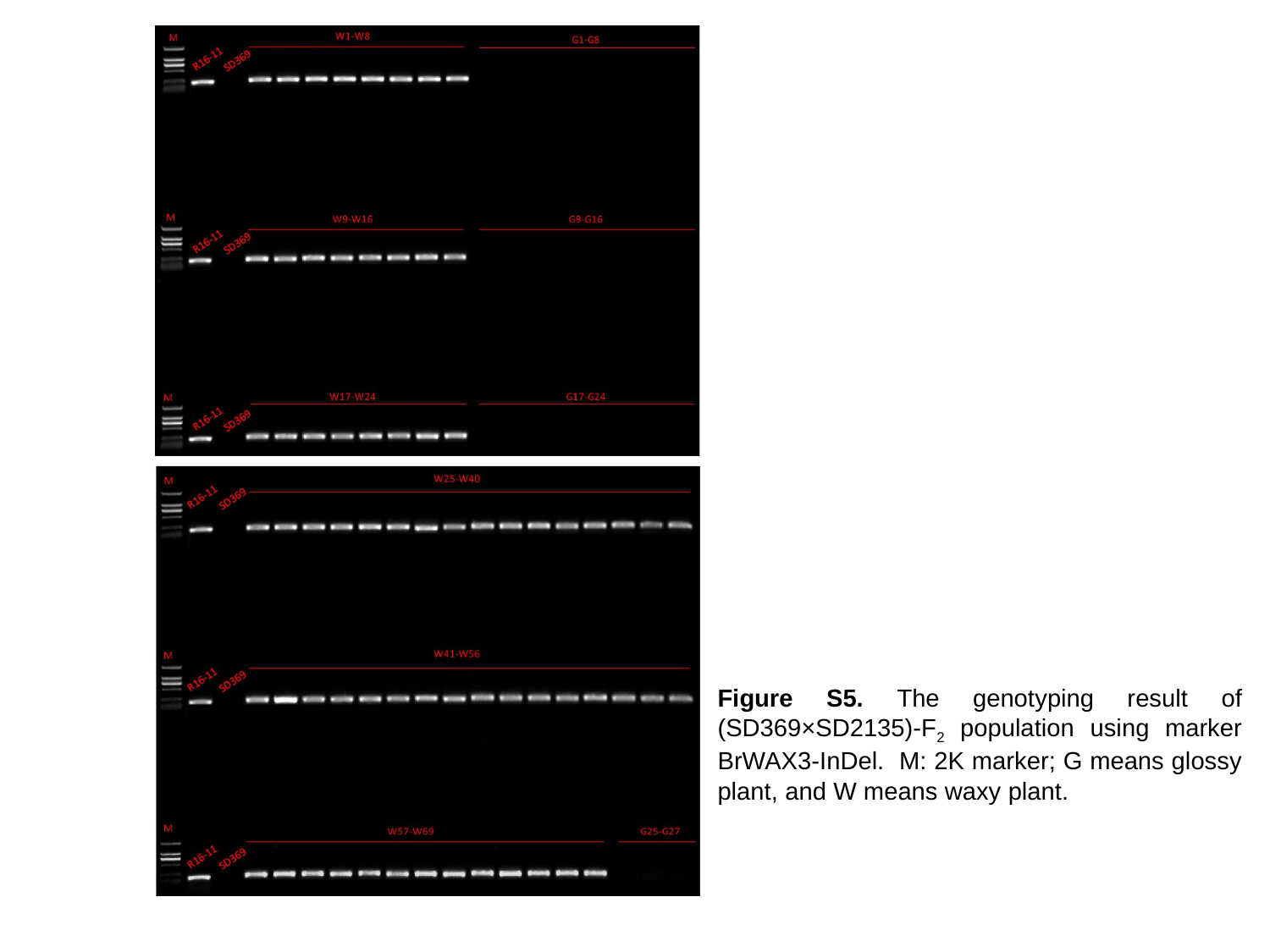

Figure S5. The genotyping result of (SD369×SD2135)-F2 population using marker BrWAX3-InDel. M: 2K marker; G means glossy plant, and W means waxy plant.

## Slide 6
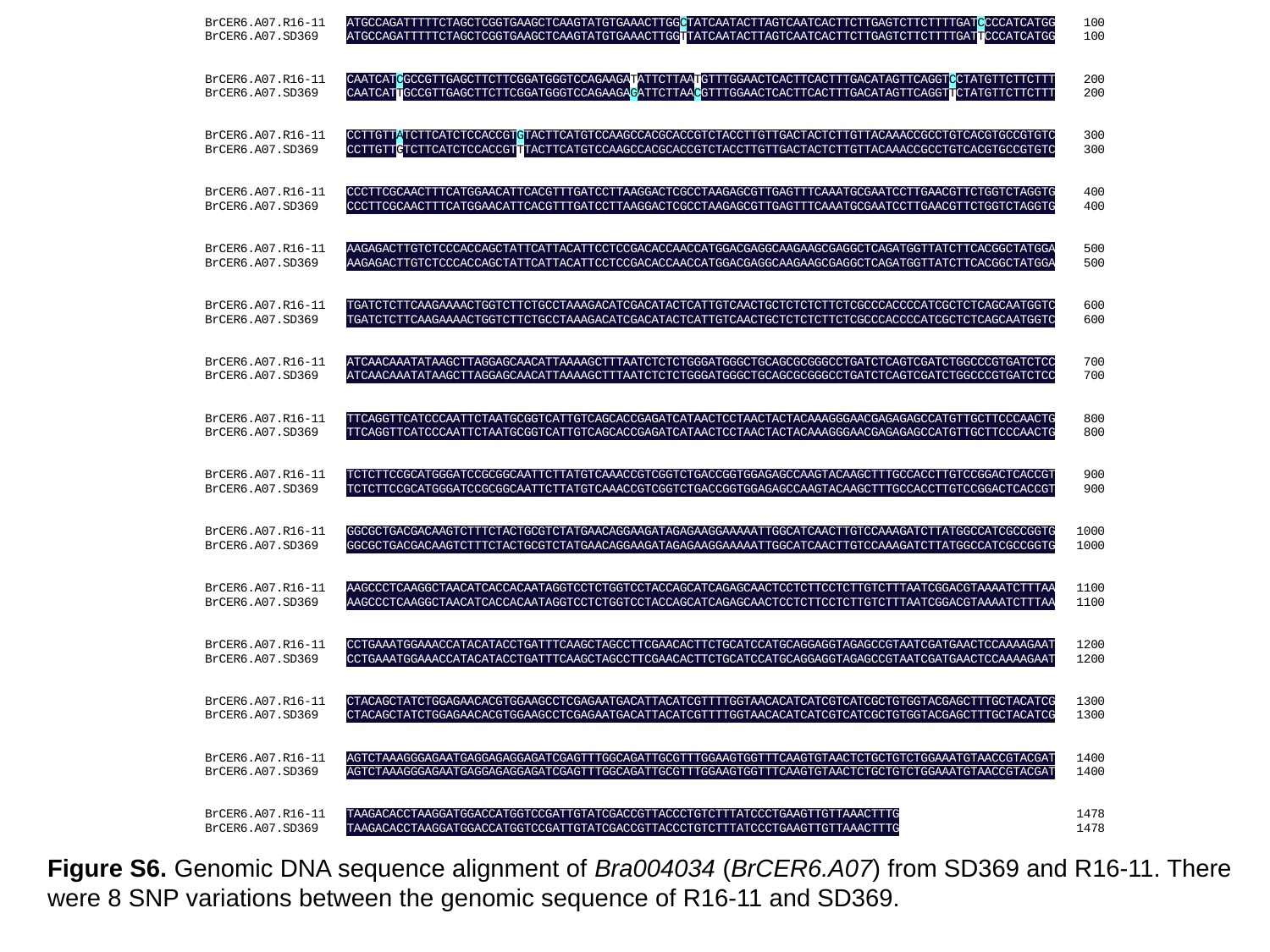

Figure S6. Genomic DNA sequence alignment of Bra004034 (BrCER6.A07) from SD369 and R16-11. There were 8 SNP variations between the genomic sequence of R16-11 and SD369.

## Slide 7
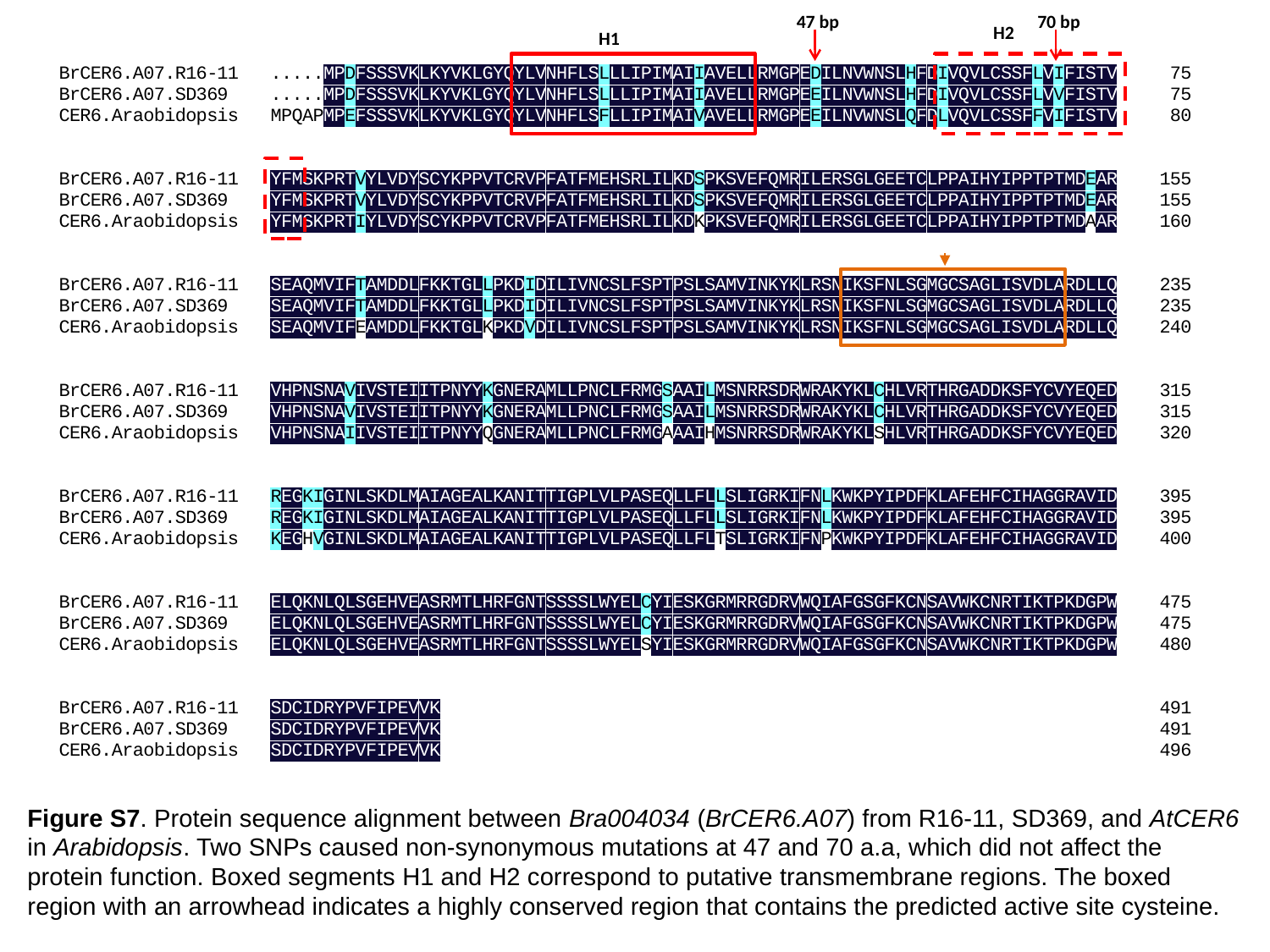

47 bp
70 bp
H2
H1
Figure S7. Protein sequence alignment between Bra004034 (BrCER6.A07) from R16-11, SD369, and AtCER6 in Arabidopsis. Two SNPs caused non-synonymous mutations at 47 and 70 a.a, which did not affect the protein function. Boxed segments H1 and H2 correspond to putative transmembrane regions. The boxed region with an arrowhead indicates a highly conserved region that contains the predicted active site cysteine.

## Slide 8
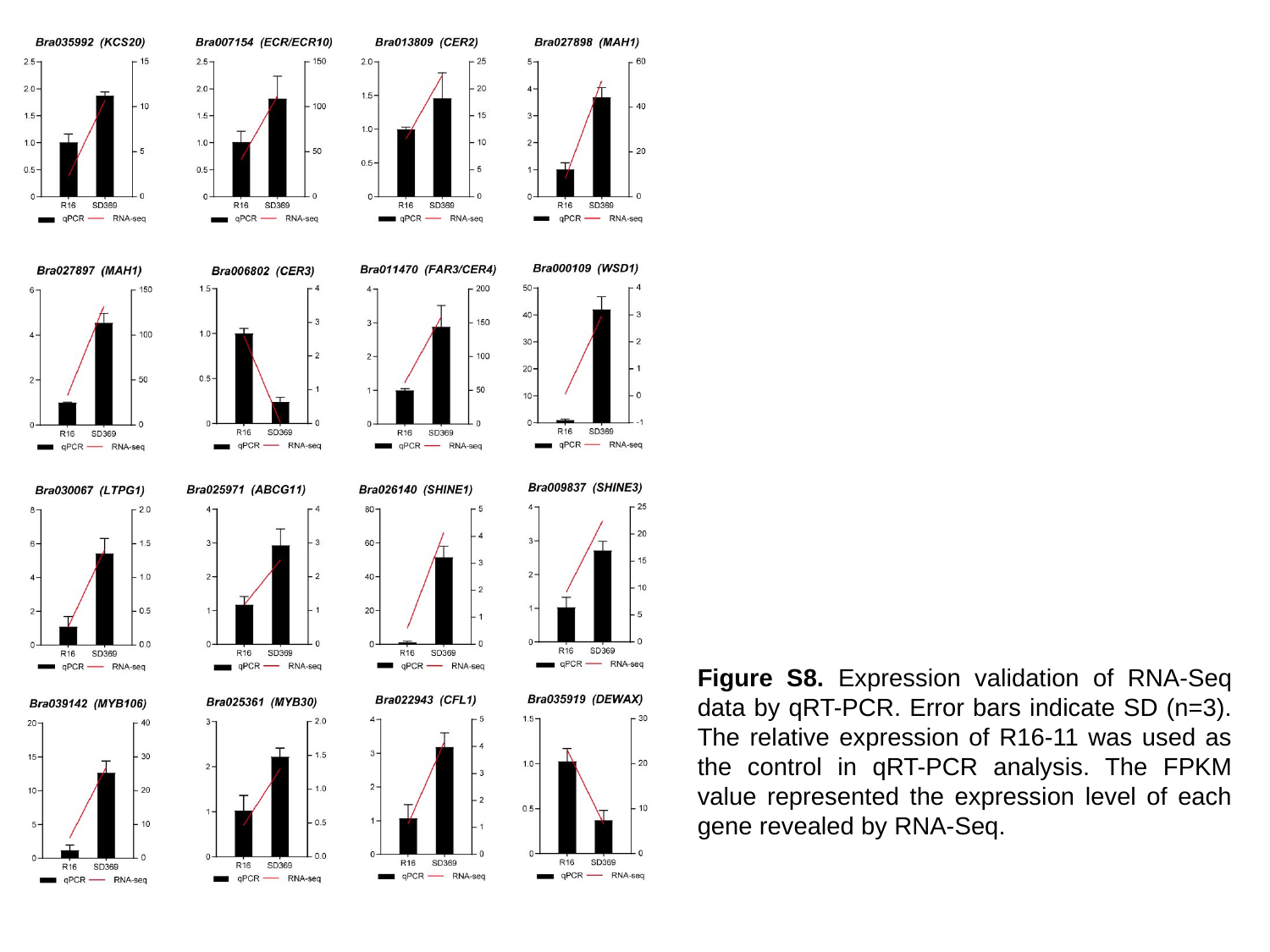

Figure S8. Expression validation of RNA-Seq data by qRT-PCR. Error bars indicate SD (n=3). The relative expression of R16-11 was used as the control in qRT-PCR analysis. The FPKM value represented the expression level of each gene revealed by RNA-Seq.

## Slide 9
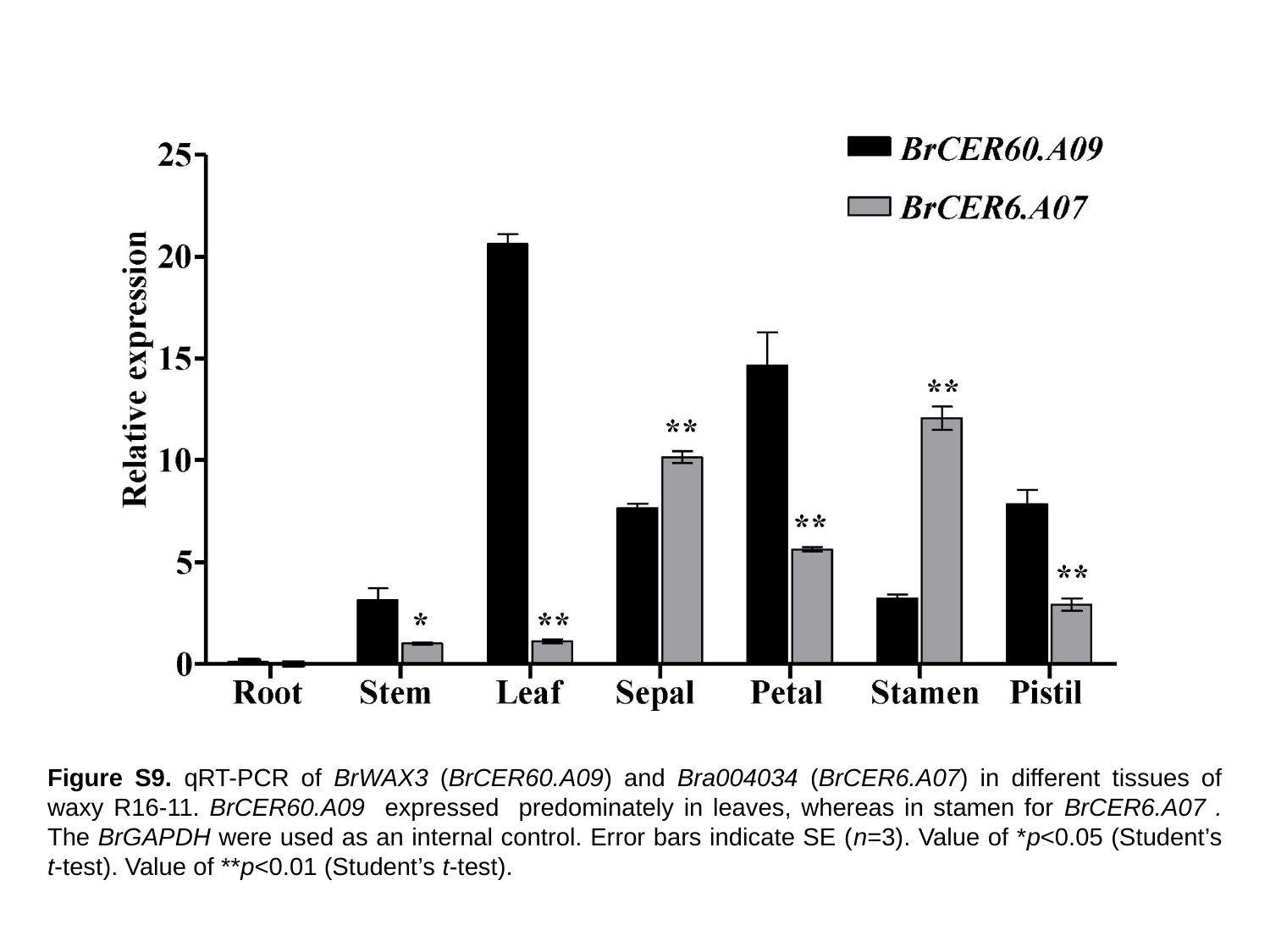

Figure S9. qRT-PCR of BrWAX3 (BrCER60.A09) and Bra004034 (BrCER6.A07) in different tissues of waxy R16-11. BrCER60.A09 expressed predominately in leaves, whereas in stamen for BrCER6.A07 . The BrGAPDH were used as an internal control. Error bars indicate SE (n=3). Value of *p<0.05 (Student’s t-test). Value of **p<0.01 (Student’s t-test).
